# Reproduction is driven by seasonal environmental variation in an equatorial mammal, the banded mongoose (*Mungos mungo*)

**DOI:** 10.1093/beheco/araf007

**Published:** 2025-01-31

**Authors:** Monil Khera, Kevin Arbuckle, Francis Mwanguhya, Michael A Cant, Hazel J Nichols

**Affiliations:** Department of Biosciences, Swansea University, Swansea, SA2 8PP, United Kingdom; Faculty of Medicine, Health and Life Science, Swansea University, Swansea, SA2 8PP, United Kingdom; Department of Biosciences, Swansea University, Swansea, SA2 8PP, United Kingdom; Banded Mongoose Research Project, Queen Elizabeth National Park, PO Box 66 Lake Katwe, Kasese District, Uganda; Centre for Ecology and Conservation, University of Exeter, Cornwall, TR10 9EZ, United Kingdom; Department of Biosciences, Swansea University, Swansea, SA2 8PP, United Kingdom

**Keywords:** climate change, food availability, *Mungos mungo*, seasonal breeding, time series decomposition, tropics

## Abstract

Reproduction is an energetically costly activity and so is often timed to occur when conditions are most favorable. However, human-induced changes in long-term, seasonal, and short-term climatic conditions have imposed negative consequences for reproduction across a range of mammals. Whilst the effect of climate change on reproduction in temperate species is well known, its effect on equatorial species is comparatively understudied. We used long-term ecological data (~20 yr) to investigate the impact of changes in rainfall and temperature on reproduction in an equatorial mammal, the banded mongoose (*Mungos mungo*). After controlling for the effects of group-size, we found that more females were pregnant and gave birth following periods of high seasonal rainfall, pregnancies increased at higher seasonal temperatures, and births increased with long-term rainfall. This is likely beneficial as high rainfall is positively associated with pup growth and survival. Females cannot, however, carry and raise pups over the course of a single wet season, so females face a trade-off in reproductive timing between maximizing resource availability during gestation or the early life of pups, but not both. Since the duration of the wet seasons is predicted to increase with climate change, the optimum conditions for banded mongoose reproduction may be extended. However, the potential benefits of extended wet seasons may be counteracted by the negative impacts of high temperatures on pup growth and survival. Our results highlight the importance of seasonality in reproduction of tropical mammals and the complex impacts of anthropogenic climate change on recruitment in equatorial species.

## Teaser

In equatorial banded mongooses we show that reproduction is highly seasonal as most females were either pregnant or gave birth during the wet seasons when food is plentiful. Our results suggest that increasing rainfall over time may help boost reproduction for some tropical mammals by increasing food availability, though this may be counteracted by negative effects of rising temperatures on offspring growth and survival.

## Introduction

The timing of reproduction is an important life history decision which impacts individual fitness and has consequences for the survival of a population over time (Milligan and Lloyd 2009). Reproduction is also energetically costly and so under poor environmental conditions, when food is scarce, it may be best to delay reproduction until conditions improve (Bronson 2009). This leads to many populations reproducing seasonally at a time of year when conditions are most favorable (Bronson 2009). Indeed, reproduction is found to be strongly linked to environmental conditions in a variety of mammalian species (Bronson 1985) from bats (Lučan et al. 2013) to bears (Spady et al. 2007).

In temperate climates, reproductive rates are usually higher during spring and summer months where high air temperatures positively affect food availability, compared to the winter months where food availability is often low (Wingfield et al. 1992). Whilst these seasonal changes in environmental conditions are usually reliable in temperate climates, favorable conditions for reproduction (such as weather conditions and food availability) are generally less predictable at lower latitudes (Bronson 1985; Wingfield et al. 1992; Brown and Shine 2006). The seasonality of the tropics has often been overlooked due to mean monthly temperatures being relatively constant compared to temperate latitudes (Janzen 1967; Abernethy et al. 2018). However, tropical climates are typically characterized by marked wet and dry seasons. So, whilst in temperate regions the timing of reproduction is strongly linked to temperature, in lower latitudes rainfall can become more important (Cohen et al. 2018). This presents a greater challenge for tropical species since they must time their reproduction in accordance with rainfall, which is comparatively less predictable both temporally and spatially than temperature (Shine and Brown 2008). Consistent with this, previous studies on tropical animal populations, including those on mandrills (*Mandrillus sphinx*) (Dezeure et al. 2022) and bats (Zortéa 2003), have found that reproduction is often timed to coincide with wet seasons because higher rainfall tends to increase food availability. Nevertheless, most previous studies on the timing of reproduction have focused on temperature-based rather than rain-based seasons (Varpe 2017).

Although many animal populations are adapted to regular and predictable seasonal changes in their external environment (Brogi et al. 2022), human-induced climate change has disrupted reproduction in many species by altering seasonal cues. For instance, multiple bird species have started migrating to breeding grounds earlier in the year (Cotton 2003; Mills 2005; Sparks et al. 2005) and reproductive success has declined in numerous species including Magellanic penguins (*Spheniscus magellanicus*) (Boersma and Rebstock 2014), northern muriquis (*Brachyteles hypoxanthus*), (Wiederholt and Post 2011) and baboons (*Papio cynocephalus*) (Beehner et al. 2006). Whilst some species such as red deer (*Cervus elaphus*) have been shown to adapt their reproductive timing to match this change in climate (Moyes et al. 2011), other species such as roe deer (*Capreolus capreolus*) have continued to give birth at the same time despite the earlier onset of spring (Plard et al. 2014). This has resulted in a temporal mismatch between the seasonal peak in births and optimum foraging conditions, with negative consequences for offspring fitness and early life survival, both at the individual and population level (Plard et al. 2014).

The impact of climate change on reproduction in tropical animal populations is arguably more difficult to predict compared to those in temperate regions since reproduction is generally more affected by rainfall (Shine and Brown 2008) and the effect of climate change on rainfall also appears to vary between regions (Hendrix and Salehyan 2012; Feng et al. 2013). Furthermore, whilst the impact of climate change on species living in mid and high latitudes has been extensively studied, the impact on equatorial species has been comparatively understudied (Feeley and Silman 2011). Long term studies on tropical populations are vital to our understanding of these questions but are challenging due to the lack of resources allocated to conducting scientific research in these regions, and to long-term studies of wild populations generally (Abernethy et al. 2018). Some previous studies on tropical species have also assumed that because there is little year-round variation in temperature, it is biologically unimportant (e.g. Marshall et al. 2016). However, since tropical animals generally have narrow thermal ranges, they could be disproportionately sensitive to even small temperature changes (Tewksbury et al. 2008; Bozinovic et al. 2011). Tropical species may therefore be particularly vulnerable to rapid climate change compared to temperate species which naturally experience greater fluctuations in temperature (Şekercioǧlu et al. 2012). The heat dissipation limit theory suggests that reproductive output in endotherms should be constrained by their ability to dissipate body heat resulting in reproduction being negatively affected by high temperatures (Speakman and Król 2010). Some support for this has been provided by studies conducted on tropical mammals, for example, increasing long-term temperatures appear to be leading to reduced fitness in wild dogs (*Lycaon pictus*) (Abrahms et al. 2022), banded mongooses (*Mungos mungo*) (Khera 2024) and meerkats (Van de Ven et al. 2020). There are, however, few other studies that have investigated the impact of rising temperatures on the reproductive success of tropical mammals.

In addition to the impact of long-term and seasonal changes in environmental conditions, short-term fluctuations, including extreme weather events, can also have significant impacts on reproduction and are expected to become more frequent as climate change continues (IPCC 2012). For example, in Argentina, not only has the higher variability in climate lowered reproductive success in Magellanic penguins (*Spheniscus magellanicus*), but as the intensity and frequency of storms continues to increase so does the likelihood of reproductive failure (Boersma and Rebstock 2014). Similarly, following periods of drought, female baboons (*Papio cynocephalus*) are less likely to enter reproductive cycles, are less likely to conceive and are less likely to carry pregnancies to term (Beehner et al. 2006). Heat-waves can also cause individuals to suffer from heat stress which can disrupt reproduction (Takahashi 2012). Individuals suffering from heat stress may reduce their food intake to slow down their metabolism and therefore reduce heat production in the body; this can impact both energy balance and nutrient availability, both of which can affect pregnancies (Hansen 2009). Finally, high short-term temperatures have also been shown to affect human reproduction during the first few weeks after conception by increasing the risk of pregnancy loss (Hajdu and Hajdu 2021). Hence, impacts of changing climates may operate through both long-term and short-term changes, and disentangling these scale-dependent effects in natural systems requires long-term life history data on wild populations.

Our 20-yr study on banded mongooses in Uganda presents an exceptional opportunity to advance our understanding of the impacts of seasonal, short- and long-term changes in environmental conditions on reproduction. First, this population has experienced variation in both rainfall and temperature over different timescales. As is typical of the tropics, the climate in Uganda is characterized by high seasonal variation in rainfall (with two wet seasons and two dry seasons per year) and low levels of seasonal variation in temperature (Marshall et al. 2016). Our study population also experiences short term fluctuations in conditions, with some months being wetter, drier, hotter or cooler than average for the time of year. Environmental conditions in Uganda are also changing over longer time periods; across western Uganda, rainfall has on average increased between 1983 and 2017, with increases in both the total amount of rainfall and the duration of wet seasons, although with considerable between-year variation (Diem et al. 2019a, 2019b). The average temperature in southwest Uganda has also increased by an average of 0.3 °C each decade since the 1960’s (GoU 2007), and temperatures are predicted to continue to rise in western Uganda and more broadly across the Great African Lakes region by 1 to 2 °C by 2050 (Babel and Turyatunga 2015; Asefi-Najafabady et al. 2018).

Second, we have collected extensive data on fecundity in our study population, allowing us to investigate the impact of variation in environmental conditions on all stages of pregnancy, from conception to birth. Banded mongooses live in social groups of approximately 10 to 30 adults, which reproduce up to four times per year and can give birth in any month, although births are not uniformly distributed over the year. Reproduction is synchronized within (but not between) groups (Hodge et al. 2011; Cant et al. 2016). Female group-members enter estrus within a few days of each other, after which a mean of 83% of adult females become pregnant, carrying up to five pups each (mean ± SD = 2.84 ± 0.12) (Cant 2000). Not all pregnancies are carried to term, with at least 43% of pregnancies being lost before birth (Inzani et al. 2019). Females give birth in close synchrony (usually on the same day) (Hodge et al. 2011) and the resultant litters are raised communally with input from most group-members (Cant 2003).

Rainfall is likely to impact on reproduction through its effect on the availability of invertebrate prey; at our study site higher annual rainfall is associated with increased invertebrate abundance (Marshall et al. 2017), and seasonality impacts the abundance of millipedes and beetles (key prey species) (De Luca 1998) influencing banded mongoose diet (Rood 1975). Moreover, these changes are associated with fitness measures; beetle and millipede abundances are positively associated with adult body condition in larger banded mongoose social groups (De Luca 1998) and increased rainfall over short periods (1 to 2 mo) is linked to increased pup mass (Nichols et al. 2012) and survival (Khera 2024). Previous studies have investigated the impact of rainfall over different time periods on banded mongoose fecundity and have found inconsistent results. Gilchrist et al (2004) found no impact of rainfall over the past 5 mo on conception probability, abortion probability, fetus count, or inter-conception interval, but there was a significant impact on age at first conception. Similarly, Cant (2000) found no significant effect of rainfall during the month of estrus, nor during the month in which females gave birth, on the proportion of females in the group breeding, and Marshall et al (2017) found no impact of mean or variation in rainfall over the first year of life on female body condition, lifespan, or lifetime reproductive success (although there were significant impacts on males). However, Nichols et al (2012) found that rainfall over the last 60 d of gestation impacted the breeding success of low-ranking, but not high-ranking, females. This was because, when rainfall was low and within-group competition for resources was high, low-ranking females were aggressively evicted from their social groups, leading them to abort their litters (Nichols et al. 2012; Inzani et al. 2019). However, we do not know whether these impacts are related to seasonal changes in rainfall, longer-term climatic trends, or short-term weather events such as droughts, making it unclear what the impacts of climate change may be on banded mongoose reproduction. Indeed, these three distinct components of rainfall variability have not previously been separated in the banded mongoose system, and variation in effects between components may have masked effects in previous work. Furthermore, no study has yet investigated the impact of temperature on fecundity in banded mongooses.

Here, we decompose variation in both rainfall and temperature into short-term, seasonal, and long-term trend components, and investigate their impact on female fecundity (pregnancy, births, and number of fetuses carried). We predict that reproductive timing will align with predictable seasonal changes in rainfall. As high rainfall leads to faster pup growth (Bell et al. 2012), females may benefit from timing their births to coincide with rainy seasons, whereby food will be most abundant for growing pups. We also predict that, due to impacting food supply, higher levels of rainfall over long periods will lead to increases in birth rates and fetus numbers, because this may lead to a reduced likelihood of fetuses being aborted. High short and long-term temperatures could cause heat stress in females, which may reduce fecundity, in which case birth rates and fetus counts may be lower when temperatures are high.

## Methods

### Study system and data collection

We used behavioral, life history and environmental data collected from a study population of wild banded mongooses in Mweya, Queen Elizabeth National Park, Uganda (0° 12’S, 27°54’E) collected between June 2000 and July 2020. At any one time, the population consisted of approximately 250 individuals, living in 10 to 12 social groups. Our study population is habituated to observation at <10 m (usually < 5 m) and groups were visited every 1 to 3 d by observers on foot to determine group composition, monitor pregnancies and determine birth dates. Individuals were identified in the field either using a unique pattern of hair dye, a unique shave pattern in their fur or, in the case of some adults, a color-coded plastic collar. All individuals were trapped and anesthetized every 3 to 6 mo so that their shave patterns, markings, and collars could be maintained (Cant 2000; Hodge 2007; Jordan et al. 2010). In brief, trapping involved capturing individuals in baited box traps placed in the shade and checked at least hourly. Once captured, traps were covered with dark cloth and were transported to the laboratory where mongooses were anesthetized using vaporized isoflurane. Once individuals had recovered from the anesthesia they were released back to their group, usually within 4 h (Jordan et al. 2010). When individuals were first captured, they were visually inspected to determine sex and were given either a uniquely coded tattoo or (since 2009) a pit tag (TAG-P-122IJ, Wyre Micro Design Ltd., UK) for permanent identification. Radio collars weighing 27g (<2% of body mass; Sirtrack Ltd., New Zealand) with a 20 cm whip antenna were attached to 1 or 2 individuals per group, allowing them to be tracked. Pregnant females were identified at around 30 to 40 d into their pregnancy due to the visible swelling of their abdomen. In many cases, this was confirmed by ultrasound scans and palpation of the abdomen, which allowed us to determine the number of fetuses that each female carried (Cant 2000; Gilchrist 2006a; Inzani et al. 2019). Births usually occurred at around 55 to 70 d after conception and were identified based on the absence of the previously pregnant females on foraging trips the morning after birth, the start of pup-care behavior, abdomen size returning to normal, and distended nipples associated with lactation (Gilchrist 2006a; Hodge et al. 2011). We defined group size as the number of individuals over 6 mo old present in the group at the relevant time point (Cant 2003; Gilchrist et al. 2004).

### Ethics statement

This study was approved by the Ethical Review Committee of Exeter University and Swansea University (210721/4401). Long-term fieldwork was approved by the Uganda Wildlife Authority (COD/96/05), Uganda National Council for Science and Technology, and adhered to the “Guidelines for the ethical treatment of nonhuman animals in behavioral research and teaching,” published by ASAB Ethical Committee/ABS Animal Care Committee (2024).

### Decomposition of environmental variables

To quantify environmental variation, we used rainfall (mm) and maximum temperature (°C) data collected from Mweya meteorological station, at the center of our study site. When modeling our environmental variables, we differentiated between short-term environmental fluctuation, seasonal variation and long-term trends (West 1997). To do this we calculated monthly averages for temperature and rainfall, and subsequently formatted our data as a time series. We then decomposed these temperature and rainfall data into three components using the *decompose* function in R 3.3.1 (R Studio Team 2016): (1) seasonal variation representing consistent intra-annual change (2) long-term trends, and (3) short-term environmental fluctuations representing irregular changes in the environment. The decompose function estimates long-term changes using moving averages, then extracts the seasonal component by calculating the average value for each month across all years. This value is then centered on the trend value. Short-term variation is the residual variation left over from the time series once the long-term and seasonal components are removed. Over the 20-yr period of our study, for logistical reasons it was not always possible to collect daily environmental data (e.g. due to staff sickness or time constraints), resulting in a small proportion of missing values: 524 d (6.75%) for maximum temperature and 421 d (5.42%) for rainfall. We imputed missing values using the imputeTS package (Moritz and Bartz-Beielstein 2017) prior to the time series decomposition. The decomposed environmental variables were used as fixed effects in our subsequent statistical models.

### Statistical analyses

We used generalized linear mixed effects models (GLMMs) implemented using the R package lme4 (Bates et al. 2015) in R 3.3.1 (R Studio Team 2016) to investigate the effects of environmental variables on all stages of pregnancy. We constructed three groups of models, each with a different fecundity-related response variable; (1) the total number of pregnancies per social group per month (2) the total number of births per social group per month and (3) the number of fetuses carried by individual females. Because groups with more adult females have a greater reproductive potential within a given month, we also investigated the proportion of females in each group that were recorded as being pregnant or had given birth. However, the proportional results differed little from the total number results, so these are presented in the supplementary material only. Our environmental variables were fitted as explanatory variables, alongside group size and group size squared to account for linear and non-linear impacts related to group size, such as intra- and inter-group competition for resources. All continuous explanatory variables were scaled and centered so that the main effects could be directly compared despite being on different scales (Schielzeth 2010). As multiple observations were taken from the same social group, we fitted group identity as a random effect, except when this resulted in a variance of zero. We fitted each model as described and did not conduct any model simplification procedures. All models were checked for over or under dispersion. The “BOBYQA” optimizer algorithm was used in our pregnancy and birth models to resolve problems with convergence (Bates et al. 2015).

It is possible that there may be a delay in the effect of our environmental variables, for example it may take time for rainfall to alter invertebrate abundance. To account for this possibility, we compared models incorporating a zero and 1-mo lag in our environmental variables using Akaike Information Criterion (corrected for sample size; AICc) (Johnson and Omland 2004). In the case where a 1-mo lag improved the model fit by more than AIC 2 points, we compared this model against a model incorporating a 2-mo lag. As a 2-mo lag did not improve the model fit by more than 2 points for any of our analyses, we did not investigate the effect of further lags in our environmental variables (Supplementary Tables S1–S5).

### Pregnancy rates

Between 2000 and 2019, we made 1641 monthly observations covering 21 social groups, with 498 of those observations (30.3%) finding at least one female in the group to be pregnant. To model the number of pregnant females (per group per month) we used GLMM’s with group identity fitted as a random effect. To correct for overdispersion (θ = 1.65), we used a negative binomial distribution.

### Birth rates

Between 2000 and 2019, we made 1861 monthly observations covering 24 social groups, with 677 of those observations (36.4%) finding at least one female in the group to have given birth. The greater number of birth observations compared to pregnancies is because some pregnancies were not directly observed due to groups not being found or temporarily moving outside of the study area. Pregnancies were also harder to detect in groups and individuals that were less well habituated to observation, whilst births are much more conspicuous due to the appearance of pups and the initiation of pup care behavior. To model the number of females that gave birth (per group per month) we used GLMMs with group identity fitted as a random effect. To correct for overdispersion (θ = 1.64) we used a negative binomial distribution.

### Number of fetuses

Between 2009 and 2013 we used 229 ultrasound scans from 93 pregnant females covering 9 social groups to determine the effects of environmental conditions and group size on the number of fetuses carried by pregnant females. When ultrasounds were taken, there was uncertainty in 9 observations (3.9%) concerning the number of fetuses present due to the size and position of the fetuses and, in these cases, ranges were given instead. If this occurred, we took the midpoint and rounded down to the nearest whole number. We modeled the number of fetuses using GLMMs with group identity and the female from which the scans were taken fitted as a random effect since scans were sometimes taken from the same individual during different pregnancies. We found that the data was underdispersed (θ = 0.530) which can result in standard errors being overestimated and biased inferences (Forthmann and Doebler 2021). We therefore used a Conway-Maxwell-Poisson distribution from the glmmTMB package (Brooks et al. 2017) which accounts for underdispersion (Shmueli et al. 2005).

## Results

### Decomposition of environmental variables

**Fig. 1.**
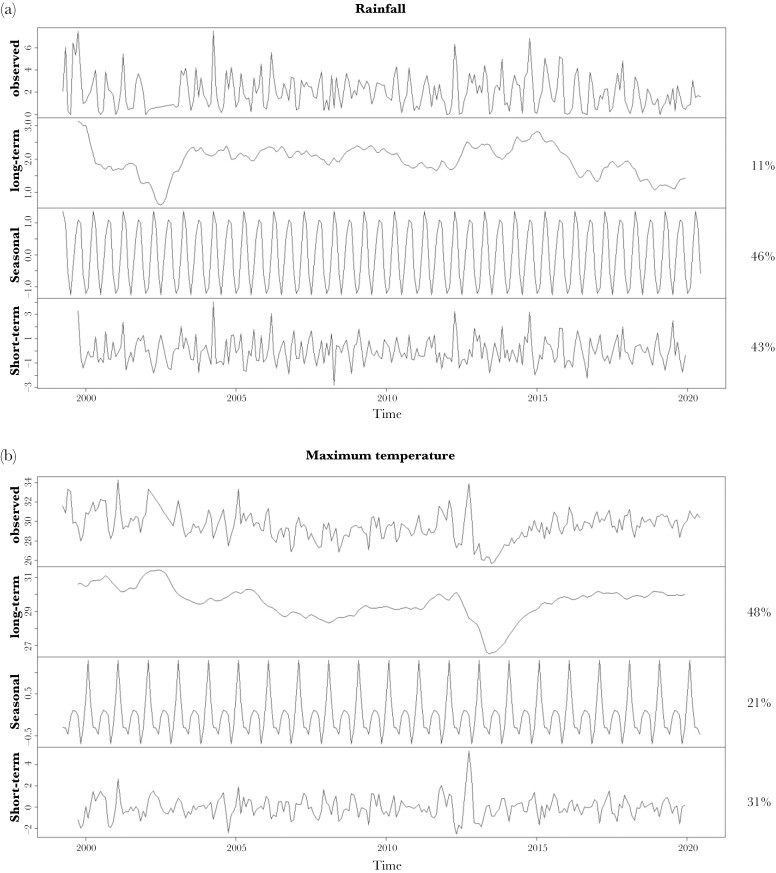
Time series decomposition of the mean daily (a) rainfall (mm) and (b) maximum temperature (°C) per month from October 1999 to December 2019 including the long-term changes, seasonal fluctuation, and short-term variation, along with the percent of variance explained by each component. The trend values are in the original scale whilst seasonality was centered on the trend value. The random variation is the residual variation left over once the trend and seasonal component are removed from the time-series.

Rainfall varied considerably between months, with observed average daily rainfall calculated monthly ranging from 0 to 7.51 mm. Our decomposition showed that seasonal variation explained a large percentage of variance (46%) in the observed average monthly rainfall (Fig. 1a); with two distinct peaks in seasonal rainfall per year, one lower but longer lasting (the long-wet season) and another higher but short lived (the short-wet season). Long-term changes explained relatively little variance (11%) in rainfall, while short-term changes explained 43% of variance, demonstrating that rainfall often fluctuates over short timescales.

Temperatures remained relatively constant over time, with the observed average daily maximum temperatures calculated monthly ranging from 25.68 to 34.29 °C. Seasonal variation in temperature was lower than for rainfall, explaining 21% of variance (Fig. 1b). Long-term changes explained almost half (48%) of the variation in the observed monthly maximum temperature while short-term changes explained 31% of the variance in temperature (Fig. 1b), demonstrating that maximum temperatures generally change more gradually over longer timescales compared to rainfall.

### Pregnancy rates

**Fig. 2.**
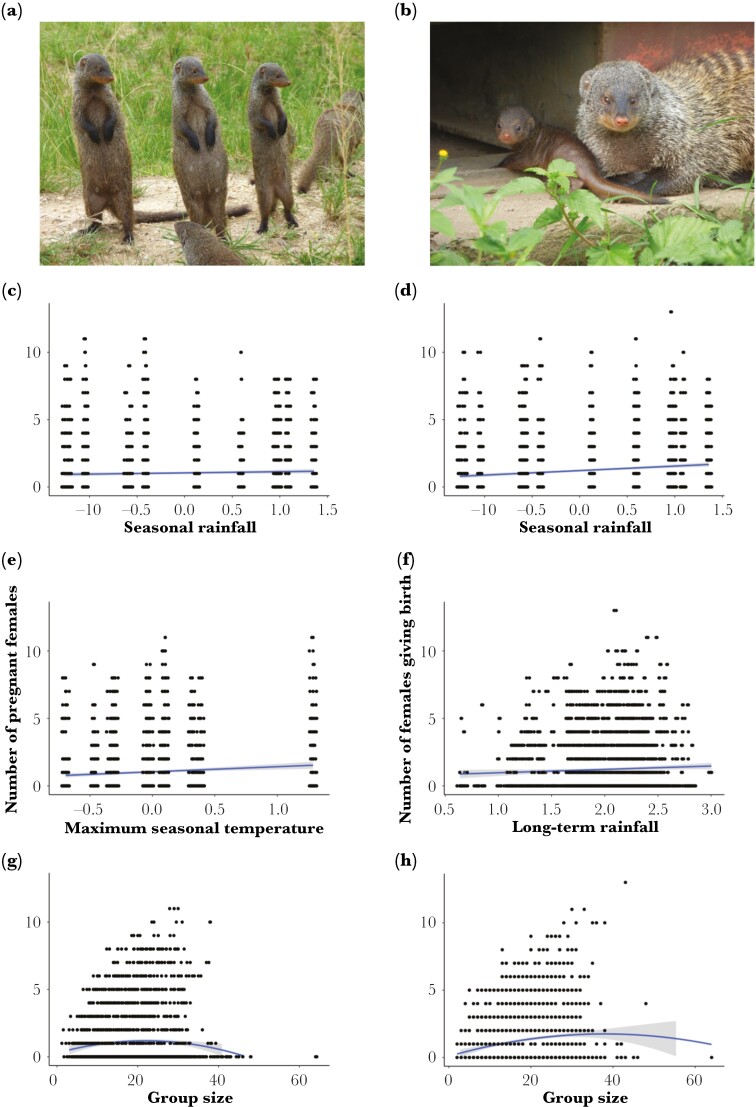
Pregnancies and births in banded mongooses. The left-hand side of the panel shows (a) pregnant females (Photo by Dr Hazel Nichols), and the number of pregnant females per group per month as a function of (c) seasonal rainfall (mm) values for the previous month, (e) maximum seasonal temperature (°C) values for the previous month and (g) group size. The right-hand side of the panel shows (b) a banded mongoose with a dependent pup shortly after birth (Photo by Dr Hazel Nichols), and the number of females giving birth per group per month as a function of (d) seasonal rainfall (mm) values for the previous month, (f) long-term seasonal rainfall (mm) values for the previous month and (h) group size. Whilst the long-term trend in rainfall (mm) is in the original scale, the seasonal variables are centered on the trend. Trend lines were fitted from the raw data based on a GLM relationship using the package “*ggplot2”* (Wickham 2016), with the shaded regions showing the 95% confidence interval.

We found a strong degree of seasonality in pregnancy rates, with more pregnancies occurring in warmer and wetter seasons (Table 1, Fig. 2, Table S6, Fig. S1). We also found a lag in the impact of environmental variables, with temperature and rainfall in the previous month having the strongest effect on pregnancy. Short- and long-term variation in environmental variables had no significant effect on pregnancy rates (Table 1, Table S6). Intermediate-sized groups had the largest number of pregnant females (Table 1, Fig. 2). This is probably because small groups contain few females, whilst large groups show greater levels of reproductive competition (Cant et al. 2001; Gilchrist 2006b). Consistent with this, the proportion of pregnant females decreased with increasing group-size (Table S6, Fig. S1).

**Table 1.** The number of pregnant females per group per month as a function of group size and environmental conditions in the previous month. Significant p-values are presented in bold.

Fixed effects	Estimate	SE	z-value	p-value
(intercept)	0.129	0.100	1.292	
Group size	0.025	0.074	0.336	0.737
Group size^2^	-0.176	0.055	-3.227	**1.25 × 10** ^**-3**^
Short-term rainfall	-0.023	0.062	-0.374	0.708
Maximum short-term temperature	-0.027	0.057	-0.478	0.632
Maximum seasonal temperature	0.302	0.067	4.502	**6.74 × 10** ^**-6**^
Seasonal rainfall	0.262	0.068	3.824	**1.31 × 10** ^**-4**^
Maximum long-term temperature	-0.010	0.067	-0.154	0.878
Long-term rainfall	0.010	0.069	0.143	0.886

### Birth rates

The number of females giving birth (per group per month) was higher during wet seasons, but did not vary with seasonal temperature (Table 2, Table S7, Fig. 2, Fig. S2). As with pregnancy rates, there was a one-month lag in the impact of environmental conditions. Additionally, we found that more females gave birth in intermediate to large sized groups (Table 2, Fig. 2). This is likely due to there being more females in larger groups, as the proportion of females giving birth did not vary with group size (Table S7). Additionally, we found little effect of short- or long-term changes in our environmental conditions, though there was a significant positive effect of long-term rainfall (Table 2, Fig. 2, Fig. S2).

**Table 2.** The number of females giving birth per group per month as a function of group size and environmental conditions in the previous month. Significant p-values are presented in bold.

Fixed effects	Estimate	SE	z-value	P-value
(intercept)	0.192	0.072	2.669	
Group size	0.328	0.057	5.748	**9.01 × 10** ^**-9**^
Group size^2^	-0.113	0.038	-2.942	**3.26 × 10** ^**-3**^
Short-term rainfall	0.060	0.049	1.229	0.219
Maximum short-term temperature	-0.052	0.0507	-1.035	0.301
Maximum seasonal temperature	0.007	0.051	0.141	0.888
Seasonal rainfall	0.323	0.056	5.824	**5.75 × 10** ^**-9**^
Maximum long-term temperature	0.070	0.057	1.246	0.213
Long-term rainfall	0.123	0.057	2.141	**0.032**

### Number of fetuses

**Fig. 3.**
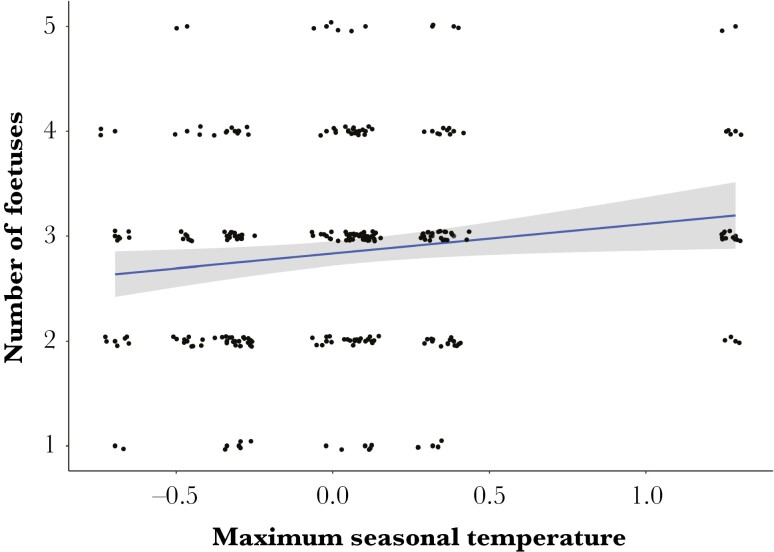
The number of fetuses identified within pregnant females during ultrasound scans as a function of the maximum seasonal temperature (°C) which was centered on the trend value. A trend line was fitted using the raw data based on a GLM relationship using the package “*ggplot2”* (Wickham 2016), with the shaded region showing the 95% confidence interval.

We found no association between rainfall and fetus number, but pregnant females carried more fetuses during warmer seasons (Fig. 3, Table 3). Fetus number was unaffected by long-term or short-term changes in rainfall and temperature (Table 3). Unlike the other fecundity-related variables, there was no lag in the effect of environmental conditions.

**Table 3.** The number of fetuses present within pregnant females as a function of group size and environmental conditions. Significant p-values are presented in bold.

Fixed effects	Estimate	SE	z-value	p-value
(intercept)	1.007	0.038	26.224	
Group size	-0.020	0.028	-0.707	0.480
Group size^2^	0.025	0.018	1.399	0.162
Short-term rainfall	-0.019	0.027	-0.706	0.480
Maximum short-term temperature	-0.043	0.027	-1.608	0.108
Maximum seasonal temperature	0.062	0.025	2.508	**0.012**
Seasonal rainfall	0.031	0.027	1.160	0.246
Maximum long-term temperature	-0.033	0.026	-1.249	0.212
Long-term rainfall	-0.024	0.029	-0.810	0.418

## Discussion

### Fecundity varies with seasonal changes in rainfall and temperature

Our study found that fecundity varied seasonally, with females being both more likely to be pregnant and to give birth following periods of high seasonal rainfall. After incorporating a 1-mo lag, in wet seasons, an average of 24.89% of females within groups gave birth each month, compared to 15% in dry seasons. Timing births to coincide with the peak of food abundance during the wet season is likely to be highly advantageous since higher rainfall increases both pup survival to emergence (Khera 2024) and pup weight (Nichols et al. 2012), and heavier pups are more likely to survive to nutritional independence (90 d) (Nichols et al. 2012). Due to the advantages for pups of being born when food is most abundant, we predicted that births would be timed to coincide with high seasonal rainfall, and our results are in accordance with this prediction.

**Fig. 4.**
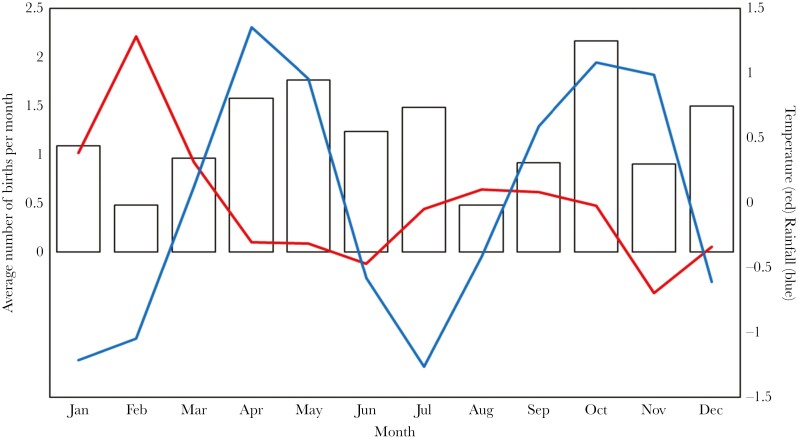
The mean number of births per group per month (bars) with seasonal rainfall (mm) (blue line) and maximum seasonal temperature (°C) (red line) overlayed. Seasonal rainfall and temperature have been centered on the trend value.

Though it is advantageous to reproduce when rainfall is high, pregnancies can last over 60 d (Cant 2000), and pups are dependent on lactation for a further 30 d and are not nutritionally independent until they are 90 d old, so it is not possible for a female to carry a pregnancy, give birth and raise the resultant litter over the course of a single wet season (which last approximately 90 d). Therefore, females that are pregnant a month after the peak of a wet season (there was a 1-mo lag in the impact of rainfall in our models) are likely to be raising their pups in the dry season, and females who give birth a month after the peak of the wet season will also likely have conceived their pregnancies during the previous dry season. The association of pregnancy and births with high seasonal rainfall therefore suggests that some pregnancies are timed so that pups are born during the rainy season, when food is most available, whilst other pregnancies are carried when food is most available, leaving the resultant litters to be raised when food is scarcer. This is illustrated by Fig. 4, where birth rates peak around the height of the wet seasons (April/May and October), but there are also secondary peaks during the early dry seasons (in July and December).

The fact that gestation and pup care extend beyond the length of a single rainy season may explain why banded mongoose reproduction is not as seasonal as one might expect; births occur in all months of the year and are not confined to the wet season. Since conception and birth are in lock-step where a substantial change in the timing of one would lead to a similar change in the timing of the other, there is a trade-off between the optimal timing of different elements of the reproductive cycle (Dezeure et al. 2021). Such a trade-off in reproductive timing, where females can either maximize seasonal resource availability when gestating their offspring or when their offspring are growing after birth (but not both) may also lead to heretofore unrecognized condition-dependent decisions over when to reproduce. For instance, if the dominant females (who appear to control the timing of reproduction within a group (Cant et al. 2014)) are in relatively poor condition then the best option for them may be to conceive during the rainy season when they can gain sufficient energy to carry the pregnancy. Whereas if dominant females are in better condition, they may be better off giving birth when resources are more available for pup growth. Alternatively, our finding that birth rates are higher during the wet seasons may be because dominant females suppress subordinate breeding when rainfall is low (and food resources are therefore likely to be in short supply) (Nichols et al. 2012). Dominant females may therefore be reproducing year-round regardless of food availability but additional births by subordinate females may occur when conditions are favorable (Nichols et al. 2012). Future studies investigating individual-level reproductive decisions will be able to investigate these hypotheses further.

Previous trends in rainfall in western Uganda from 1983 to 2017 show that the first wet season (March to May) has been extended by 27 d and has experienced a 71% increase in rainfall (Diem et al. 2019b). If this trend continues, the timeframe under which banded mongooses can reproduce under optimum conditions may also increase. Depending on how much the duration of the wet seasons extends by in the future this could potentially reduce constraints on females, allowing them to time reproduction so that they are able to experience high resource availability for a greater proportion of each breeding attempt. In combination with the limited effects of non-seasonal temperature and rainfall on reproduction, this suggests the unexpected possibility that climate change could plausibly increase banded mongoose fecundity. However, high rainfall can lead to the flooding of birthing dens, and previous work found a negative impact of high temperatures on pup survival (Khera 2024) and weight (Khera et al. 2023). These changes may counteract the positive impact of increasing rainfall under future climate change.

We found that high seasonal temperatures were positively associated with pregnancy rates and the number of fetuses carried by the mother. Females that conceive shortly after the increase in temperature early in the year (February) give birth at the peak of rainfall (April/May), while those that conceive after the increase in temperature later on in the year (August/September) give birth at the peak of the second rainy season (Oct/Nov). Changes in temperature are a well know cue for reproduction in a number of temperate bird species (Visser et al. 2009; Schaper et al. 2012; Martin et al. 2020) but have rarely been shown to be a cue for equatorial mammals (Heideman and Bronson 1994). While we cannot exclude the possibility that higher temperatures are simply correlated with other variables that relate to fecundity, it is possible that this represents a rare example of an equatorial mammal using small changes in temperature to predict future food availability for reproduction. The types of cues used by seasonally breeding equatorial mammals that cannot rely on changes in photoperiod to predict increases in food availability have received very little attention previously.

### Little to no effect of long- and short-term environmental changes on reproduction

We predicted that, due to impacting food supply, long-term periods of high rainfall would lead to an increase in birth (but not necessarily pregnancy) rates, and fetus numbers, whereby high levels of rainfall may lead to a reduced likelihood of fetuses being aborted (Nichols et al. 2012). In line with this prediction, we found that long-term periods of high rainfall slightly increased birth rate, but we did not find a similar impact on pregnancy rates. Contrary to our prediction, we found no impact of long-term rainfall on the number of fetuses carried. This suggests that longer-term change in resource availability does not affect fecundity over and above its effect on birth rates.

We also predicted that high short-term temperatures would cause heat stress in females, which may reduce fecundity. However, we found no evidence that pregnancy rates, birth rates or fetus counts were lower when short-term temperatures were high. This could be because banded mongooses behaviorally thermoregulate under high temperatures by reducing activity levels and resting more (Khera 2024), which may to some extent buffer against abnormally high temperatures. Overall, our study suggests that banded mongooses are generally resilient to short-term and long-term changes in climate with regards to the stages of reproduction we investigated. Furthermore, current evidence suggests that the region of Uganda that our study population is found in is going to experience wetter (Diem et al. 2019a, 2019b) and warmer (Babel and Turyatunga 2015; Asefi-Najafabady et al. 2018) conditions over time. Although our results suggest that these conditions may have little impact on banded mongoose fecundity (though birth rates may increase slightly), increases in long-term temperatures and rainfall that extend outside of the range experienced in our data may have unpredicted but significant impacts. Furthermore, high temperatures associated with future climate change may depress recruitment through reducing pup growth and survival (Khera 2024).

## Conclusion

Our study finds that although banded mongooses give birth year-round, they are able to time reproduction to match peak environmental conditions for either gestation or births (but not both) by either carrying pregnancies during the dry season when food is more limited but giving birth during the wet season when food is abundant, or vice versa. If wet seasons become longer over time, females may be able to conceive and give birth over the course of a single wet season, which has the potential to increase fecundity and to concentrate breeding into wet seasons. Seasonality is therefore important for reproduction in these equatorial mammals, though its impact may change over time with anthropogenic-induced climate change. We also find potential evidence of females using high seasonal temperatures as a cue to enter estrus resulting in births occurring when food availability is high. This may provide some insights into the reproductive cues used by equatorial species. Finally, although extrapolating to future climates beyond the scale of the data may be problematic, pregnancy and birth rates of banded mongooses may increase under the warmer and wetter conditions that characterize climate change in this region. However, such increases may be counteracted by increased pup mortality (Khera 2024).

## Supplementary Material

araf007_suppl_Supplementary_Tables_S1-S7_Figures_S1-S2

## Data Availability

Analyses reported in this article can be reproduced using the data provided by Khera et al. (2025).
