# Supplementary material for: Reproduction is driven by seasonal environmental variation in an equatorial mammal, the banded mongoose (Mungos mungo)
Source: Behav Ecol. 2025 Jan 31;36(2):araf007. doi: 10.1093/beheco/araf007 (PMC11840749; doi:10.1093/beheco/araf007)
Supplement: araf007_suppl_Supplementary_Tables_S1-S7_Figures_S1-S2 [file araf007_suppl_supplementary_tables_s1-s7_figures_s1-s2.docx]

**Reproduction is driven by seasonal but not short or long-term environmental variation in an equatorial mammal; the banded mongoose**

**Supplementary information on the analysis of lags in the effects of our environmental variables and on the proportion of females pregnant and giving birth**

**Statistical analysis**

We modelled the proportion of females pregnant or giving birth in each group using the cbind function in R 3.3.1 (R Studio Team 2016). We based the proportion on the number of females over 9 months old within the group, since this is the age at which females can start to reproduce (Gilchrist et al. 2004). For these models we also corrected for overdispersion for the both the pregnancy (θ = 1.91) and birth models (θ = 1.90) by adding an observation level random effect (Harrison 2015). Once the observation level random effect was added group identity explained zero (or close to zero) variation and resulted in a singular fit, so we did not include group identity in the proportion models. We then checked for any lags in the effect of our environmental variables following the same procedure outlined in the main text. As with the previous birth and pregnancy models, we found a 1-month lag in the effect of environmental variables.

**Model comparison tables for pregnancy rates**

Table S1. Model comparison table showing the competing models for the number of pregnant females analysis including models with no lag (M0), 1-month (M1), and 2-month (M2) lags in our environmental conditions. This includes the name of the model, the number of parameters (K), logLik (log-likelihood), AICc, deltaAICc (the difference between the best model and every other model), Weight (model probabilities) and Evidence ratio (the amount of evidence for the best model relative to each model i.e. a score of 2 means that there is 2 times less evidence supporting it than the best model).

| **Model** | **K** | **logLik** | **AICc** | **deltaAICc** | **Weight** | **Evidence ratio** |
| --- | --- | --- | --- | --- | --- | --- |
| M1 | 11 | -2065.457 | 4153.076 | 0.000 | 0.838 | 1.000 |
| M0 | 11 | -2067.361 | 4156.885 | 3.809 | 0.125 | 6.716 |
| M2 | 11 | -2068.577 | 4159.317 | 6.241 | 0.037 | 22.658 |

Table S2. Model comparison table showing the competing models for the proportion of pregnant females analysis including models with no lag (M0), 1-month (M1), and 2-month (M2) lags in our environmental conditions. This includes the name of the model, the number of parameters (K), logLik (log-likelihood), AICc, deltaAICc (the difference between the best model and every other model), Weight (model probabilities) and Evidence ratio (the amount of evidence for the best model relative to each model i.e. a score of 2 means that there is 2 times less evidence supporting it than the best model).

| **Model** | **K** | **logLik** | **AICc** | **deltaAICc** | **Weight** | **Evidence ratio** |
| --- | --- | --- | --- | --- | --- | --- |
| M1 | 10 | -1763.478 | 3547.090 | 0.000 | 0.647 | 1.000 |
| M0 | 10 | 1764.454 | 3549.043 | 1.952 | 0.244 | 2.654 |
| M2 | 10 | -1765.259 | 3550.654 | 3.564 | 0.109 | 5.940 |

**Model comparison tables for birth rates**

| **Model** | **K** | **logLik** | **AICc** | **deltaAICc** | **Weight** | **Evidence ratio** |
| --- | --- | --- | --- | --- | --- | --- |
| M1 | 11 | -2605.956 | 5234.054 | 0.000 | 0.901 | 1.000 |
| M2 | 11 | -2608.199 | 5238.542 | 4.487 | 0.096 | 9.428 |
| M0 | 11 | -2611.625 | 5245.393 | 11.339 | 0.003 | 289.830 |

Table S3. Model comparison table showing the competing models for the number of births analysis including models with no lag (M0), 1-month (M1), and 2-month (M2) lags in our environmental conditions. This includes the name of the model, the number of parameters (K), logLik (log-likelihood), AICc, deltaAICc (the difference between the best model and every other model), Weight (model probabilities) and Evidence ratio (the amount of evidence for the best model relative to each model i.e. a score of 2 means that there is 2 times less evidence supporting it than the best model).

Table S4. Model comparison table showing the competing models for the proportion of births analysis including models with no lag (M0), 1-month (M1), and 2-month (M2) lags in our environmental conditions. This includes the name of the model, the number of parameters (K), logLik (log-likelihood), AICc, deltaAICc (the difference between the best model and every other model), Weight (model probabilities) and Evidence ratio (the amount of evidence for the best model relative to each model i.e. a score of 2 means that there is 2 times less evidence supporting it than the best model).

| **Model** | **K** | **logLik** | **AICc** | **deltaAICc** | **Weight** | **Evidence ratio** |
| --- | --- | --- | --- | --- | --- | --- |
| M2 | 10 | -2400.066 | 4820.251 | 0.000 | 0.508 | 1.000 |
| M1 | 10 | -2400.099 | 4820.316 | 0.065 | 0.492 | 1.033 |
| M0 | 10 | -2408.729 | 4837.578 | 17.327 | 0.000 | 5786.310 |

**Model comparison table for number of foetuses**

Table S5. Model comparison table showing the competing models for the number of foetuses analysis including models with no lag (M0), 1-month (M1), and 2-month (M2) lags in our environmental conditions. This includes the name of the model, the number of parameters (K), logLik (log-likelihood), AICc, deltaAICc (the difference between the best model and every other model), Weight (model probabilities) and Evidence ratio (the amount of evidence for the best model relative to each model i.e. a score of 2 means that there is 2 times less evidence supporting it than the best model).

| **Model** | **K** | **logLik** | **AICc** | **deltaAICc** | **Weight** | **Evidence ratio** |
| --- | --- | --- | --- | --- | --- | --- |
| M0 | 12 | -291.125 | 607.694 | 0.000 | 0.822 | 1.000 |
| M1 | 12 | -292.652 | 610.747 | 3.054 | 0.179 | 4.603 |

**Results**

**Pregnancy rates**

Table S6. The proportion of pregnant females (out of the total number of females over 9 months) per group per month as a function of group size and environmental conditions in the previous month. Significant p-values are presented in bold.

| **Fixed effects** | **Estimate** | **SE** | **z-value** | **p-value** |
| --- | --- | --- | --- | --- |
| (intercept) | -7.461 | 0.308 | -24.252 |  |
| Group size | -0.775 | 0.150 | -5.153 | **2.57x10^-7^** |
| Group size^2 | 0.050 | 0.119 | 0.419 | 0.675 |
| Short-term rainfall | -0.002 | 0.148 | -0.011 | 0.991 |
| Maximum short-term temperature | -0.035 | 0.153 | -0.226 | 0.821 |
| Maximum seasonal temperature | 0.471 | 0.167 | 2.815 | **4.87x10^-3^** |
| Seasonal rainfall | 0.382 | 0.165 | 2.323 | **0.020** |
| Maximum long-term temperature | 0.008 | 0.177 | 0.046 | 0.963 |
| Long-term rainfall | -0.085 | 0.176 | -0.485 | 0.628 |

Figure S1. The proportion of pregnant females per group per month as a function of a) maximum seasonal temperatures, b) seasonal rainfall and c) group size. The seasonal variables were centered on the trend. Trend lines were fitted from the raw data based on a GLM relationship using the package ‘*ggplot2’* (Wickham 2016), with the shaded regions showing the 95% confidence interval.

**Birth rates**

Table S7. The proportion of female giving birth (out of the total number of females over 9 months) per group per month as a function of group size and environmental conditions in the previous month. Significant p-values are presented in bold.

| **Fixed effects** | **Estimate** | **SE** | **z-value** | **P-value** |
| --- | --- | --- | --- | --- |
| (intercept) | -4.209 | 0.254 | -16.568 |  |
| Group size | -0.087 | 0.113 | -0.766 | 0.444 |
| Group size^2 | -0.043 | 0.083 | -0.515 | 0.607 |
| Short-term rainfall | 0.191 | 0.116 | 1.653 | 0.098 |
| Maximum short-term temperature | -0.197 | 0.113 | -1.743 | 0.081 |
| Maximum seasonal temperature | -0.018 | 0.119 | -0.153 | 0.878 |
| Seasonal rainfall | 0.753 | 0.123 | 6.111 | **9.89x10^-10^** |
| Maximum long-term temperature | 0.136 | 0.130 | 1.046 | 0.295 |
| Long-term rainfall | 0.190 | 0.131 | 1.449 | 0.147 |

Figure S2. The proportion of females giving birth per group per month as a function of seasonal rainfall in the previous month. Trend lines were fitted using the raw data based on a GLM relationship with the shaded regions showing the 95% confidence interval.
